# 2021 Acknowledgment of *mSphere Ad Hoc* Reviewers

**DOI:** 10.1128/msphere.00957-21

**Published:** 2021-12-22

**Authors:** Michael J. Imperiale

**Affiliations:** University of Michigan Medical School, Ann Arbor, Michigan, USA

## EDITORIAL

It’s that time of year, as we head into the end-of-year holidays, to reflect on how the past 12 months have played out at *mSphere*. I would like to begin with a tremendous round of applause for all the authors, reviewers, and members of our editorial team who have continued to advance our knowledge of the microbial sciences during our second year of the COVID-19 pandemic.

I alluded to the following in last year’s message but would like to drive it home again, with the advantage of some hindsight. In early 2020, very few people would have predicted that by the end of the year we would have had an effective vaccine against SARS-CoV-2, much less multiple versions. Yet, we accomplished it, in no small part due to years of fundamental research on how the immune system recognizes and responds to pathogens, how mRNAs are synthesized and translated, and how to effectively deliver macromolecules into cells. Much of this progress was anchored in the microbial sciences: studies of viral immunology, landmark work on mRNA metabolism in bacteria and virally infected cells, understanding how pathogens bind to and enter cells, development of techniques that were originally used to introduce viral oncogenes into mammalian cells in culture, and the use of viral vectors for gene therapy all set the stage for the COVID-19 vaccines. I would be remiss if I failed to note that this research was performed all around the world.

At *mSphere*, we are committed to continuing to publish important studies that advance our knowledge of the plethora of microbes and how they interact with their hosts. I have no doubt that this research will pay enormous benefits in the future.

Our submission numbers remain high and, despite a small increase in our time to first decision late last year and early this year, we have turned that curve around and aspire to get our decision-making speed back to where it was in the “Before Times.”

I want to give special mention to the staff at ASM who have worked tirelessly to keep the gears of publication well oiled. In particular, Nikki Glenn, Amanda Donaldson, and Noel Lin have made our jobs as editors easier and delightful.

Finally, we list below the many individuals who served as *ad hoc* reviewers in 2021. They have our gratitude. I hope that you are able to enjoy some time over the next few weeks connecting or reconnecting with your families and loved ones, this year in person. Humans are social animals, and it’s great to be able to resume these activities.

Kjersti Aagaard
Mohamed M. H. Abdelbary
Sabrina Absalon
Michael C. Abt
Mark D. Adams
Josephine Azikuru Afema
Kayode Olayinka Afolabi
Surya D. Aggarwal
Hector Aguilar-Carreno
Christian Paul Ahearn
Brian M. M. Ahmer
Mustafa Akkoyunlu
Md. Tauqeer Alam
Ashraf Al Ashhab
M. John Albert
Anoop Alex
Caroline Alfieri
Holly M. Scott Algood
Jonathan Allen
Emma Allen-Vercoe
Juan C. Alonso
Francis Alonzo
Christopher Alteri
John Alverdy
Christopher S. Anderson
Matthew Zack Anderson
David R. Andes
Laura Maria Andrade De Oliveira
Marco Andreolli
Diego O. Andrey
Alberto Antonelli
Yoshiteru Aoi
Cristian Apetrei
Chelsie Elizabeth Armbruster
Sandra K. Armstrong
Jennifer M. Auchtung
Tatjana Avšič-Županc
Domenico Azarnia Tehran
Sophie Bachellier-Bassi
Michael A. Bachman
Steffen Backert
Matthew Baideme
Camden R. Bair
Jonathon L. Baker
Katherine H. Baker
Scott Baliban
Jimmy D. Ballard
Guilia Bandini
Fernando Baquero
Noa Barak-Gavish
Joseph T. Barbieri
Brianne Barker
Jason C. Bartz
Martine Bassilana
Christine Marie Bassis
Tilman Baumstark
Marco Becherelli
Sara Beier
Daniel P. Beiting
Georgios N. Belibasakis
Aeriel D. Belk
Samantha L. Bell
Jessica A. Belser
Jorge L. Benach
Jose A. Bengoechea
Peter Bergholz
Teresa M. Bergholz
Tanja Berić
David Bernstein
Stefan Bertilsson
Ralph Bertram
Sanchita Bhadra
Dipankar Bhattacharyya
Bijit Bhowmik
Fadil A. Bidmos
Claire H. Birkenheuer
Jacob P. Bitoun
Daniel Blanco-Melo
Jon S. Blevins
Joseph M. Bliss
Patricia Pringle Bloom
Antje Blumenthal
Kasun H. Bodawatta
Pierre Bogaerts
Gregory Bonito
Sébastien Bontemps-Gallo
Angela Bordin
Jens Bosse
Anna Both
Travis Bourret
Kate Bowerman
Eric Boyd
Ethna Fidelma Boyd
Todd Bradley
Rita Branco
Kyndall Braumuller
Linda Breeden
Mathieu Brochet
Nichole A. Broderick
Christopher B. Brooke
Grayson Brown
Jeremy S. Brown
Kevin M. Brown
Michael G. Brown
Harry Brumer
Donald A. Bryant
Alison Buchan
Lori L. Burrows
Karen Bush
Andrea Cabibbe
Laty A. Cahoon
Yi Cai
Eloiza Helena Campana
Edgar I. Campos-Madueno
Eric Caragata
Alessandra Carattoli
Franck Gael Carbonero
Miguel Carda Diéguez
Jeffrey Carey
Ryan B. Carnegie
Jaime Carrasco
Vern B. Carruthers
Leslie S. Casey
Irene Castano
Santiago Castillo-Ramírez
Clayton C. Caswell
Rodrigo Cayô
Daniel Cazares
Brandi N. Celia
Nuno Cerca
Miguel Angel Cevallos
Dipshikha Chakravortty
Douglas L. Chalker
Thomas M. Chambers
Josephine R. Chandler
Michael Chandler
Robert L. Charlebois
Sujata S. Chaudhari
Neeraj Chauhan
Damien Chaussabel
Michael S. Chaussee
Francisco P. Chavez
Liang Chen
Chiuping Cheng
Rachel A. Cheng
Laurent Roberto Chiarelli
Alex W. H. Chin
Michaelle Chojnacki
Stephen A. Clark
Erika C. Claud
David W. Cleary
Sara Clohisey
Shira Milo Cochavi
Darrell Cockburn
Ashley Cohen
Sean Conlan
Laura Cook
Gretchen Cooley
Brendan Cormack
Pierre Cornelis
Caitlin Cossaboom
Siobhan C. Cowley
Robert A. Cramer
Max Cravener
Alison K. Criss
Karissa L. Cross
Robert W. Cross
Liwang Cui
Paul J. Cullen
Natacha Cuoto
Cameron R. Currie
Todd Andrew Cutts
Dennis G. Cvitkovitch
F. Heath Damron
Ajai A. Dandekar
Stephen Daniels
Biswadip Das
Bryan W. Davies
Charles R. Dean
Jean-Winoc Decousser
Elizabeth N. De Gaspari
Miranda De Graaf
Kirk W. Deitsch
Harry P. De Koning
Frank R. DeLeo
Thomas G. Denes
David W. Denning
Rajendar Deora
Cynthia Ann Derdeyn
Steven C. Derrick
Jigar V. Desai
Lalitagauri Deshpande
Sanjay Kumar Dey
Vijaykrishna Dhanasekaran
Rishu Dheer
Robert P. Dickson
Diego G. Diel
Beatriz Diez Moreno
Stephen P. Diggle
Joseph P. Dillard
Siyuan Ding
Marc S. Dionne
Alan Angelo Dispirito
Dirk P. Dittmer
Eunsoo Do
Carlota Dobaño Lazaro
Yohei Doi
Janet Donaldson
Caihong Dong
Matthew J. Dorman
Laurent Dortet
Benoît Doublet
Charles M. Dozois
Jan Felix Drexler
Yuchun Du
Elves Duarte
Edward G. Dudley
Breck A. Duerkop
Anne K. Dunn
Sanjucta Dutta
Kathryn Eaton
Leo Eberl
Kathryn M. Edenborough
Tom Edlind
Elizabeth A. Edwards
Maren Eggers
Sabine Ehrt
Patrick Eichenberger
Waldir P. Elias
Jeremy R. Ellermeier
Roland Elling
Najib M. El-Sayed
Mostafa S. Elshahed
Joanne B. Emerson
Virve Irene Enne
Eeva Liisa Eronen-Rasimus
Alice L. Erwin
Javier Antonio Escobar-Perez
Matthew J. Evans
Franziska Faber
Robert Fagan
Christina S. Faherty
Linda Falgenhauer
Séamus Fanning
Mauricio J. Farfan
Matthew L. Faron
Amy K. Feehan
Mario F. Feldman
Jinrong Feng
J. Christopher Fenno
David J. Ferguson
Isabel Fernández Escapa
Astrid Ferrer
Richard L. Ferrero
Kenneth A. Fields
Joshua Fierer
Sergio R. Filipe
Maria F. Fillat
Scott G. Filler
Douglas K. Fischer
Carlos Flores
Stephanie Flowers
Fabrizio Foieni
Steven L. Foley
Laura Ford
Jarrod R. Fortwendel
Michael T. France
Kristi L. Frank
Natalia Freund
Georg Fritz
Inga Fröding
Takasuke Fukuhara
Marta M. Gaglia
Hannah Gaimster
Raj Gaji
James E. Galen
Markus Ganter
Michael G. Ganzle
Erin C. Garcia
Sarahi L. Garcia
Amy Shirley Gargis
Kathleen Gärtner
Caroline Attardo Genco
Noel Gerald
Carmen Gherasim
Lorenzo Giacani
Heather L. Glasgow
Oleg Glebov
Erin S. Gloag
Marek Gniadkowski
Richard V. Goering
Gustavo H. Goldman
Jonathan Louis Golob
Benjamin Golomb
Laura Gómez-Consarnau
Angela Gomez-Simmonds
Yanhai Gong
Jesus Gonzalo-Asensio
Steven D. Goodman
Tobias Goris
Morgan Gorris
Ria Goswami
Matthias Gotte
Revathi Govind
Manish Goyal
Andreas Grabrucker
Lisa Gralinski
Luke R. Green
Alexander L. Greninger
Finn Grey
Elizabeth Grice
Dennis Grogan
Elisabeth Grohmann
Trudy H. Grossman
Cassandra Guarino
Marc-Jan Gubbels
Eric Guédon
Pascale S. Guiton
Arda Gulay
Ravindra Kumar Gupta
Gabriel Gutkind
David Hackstadt
Andrea Hahn
Anders P. Hakansson
Riley Hale
Vanessa L. Hale
Robert Hall
Roy A. Hall
Ruth M. Hall
Brian K. Hammer
Tobin Hammer
Abdul N. Hamood
Axel Georg Hamprecht
Ken-Ichi Hanaki
Lynn E. Hancock
Blake M. Hanson
Mingju Hao
Md. Manjurul Haque
Sohei Harada
Clare Harding
Lee H. Harrison
Oliver Harschnitz
Erica M. Hartmann
Eric T. Harvill
Asma Hatoum-Aslan
Ben M. Hause
Margo G. Haygood
Cynthia Y. He
Susu He
Aoife T. Heaslip
Nicholas S. Heaton
Nagendra R. Hegde
Christine Heilmann
Henry S. Heine
David E. Heinrichs
Peera Hemarajata
Tory A. Hendry
Cristina Herencias
Ana Hernandez Cordero
Robert L. Hettich
Andrés Hidalgo
Steven Higgins
Penelope Higgs
Takahiro Hiono
Itaru Hirai
Theresa D. Ho
Thomas Hoenen
Nicole A. Hoff
Deborah A. Hogan
Peiying Hong
Lauren Michelle Hook
Thomas Hooven
Alexander M. Horspool
Paul A. Hoskisson
Daniel K. Howe
Gongzheng Hu
Ke Hu
Linden T. Hu
Stephen S. H. Huang
Eili Huhtamo
Lewis Hun
Jason F. Huntley
Jillian H. Hurst
Bonnie L. Hurwitz
Wilhelmina Huston
Justin Hutchison
Albina Ibrayeva
Melissa Ingala
Thomas J. Inzana
William W. Ja
Mary Ann Jabra-Rizk
Cody B. Jackson
Anna C. Jacobs
William R. Jacobs
Guilhem Janbon
Ingmar Janse
Michael A. Jarvis
Vicki Jeffers
Niuniu Ji
Dong-Yan Jin
William Johnson
Susan Joseph
Lok R. Joshi
Yuan Jun
Barbara C. Kahl
Maria Kalamvoki
Suzanne R. Kalb
Jeremy Phillip Kamil
Manabu Kanno
Fathi Karouia
Anbu Kumar Karuppannan
Fatah Kashanchi
Joseph Keane
Daniel B. Kearns
Scott P. Keely
Eliisa Kekäläinen
Brendan Kelly
Volkhard A. J. Kempf
Arnaud Kengmo Tchoupa
Nemat O. Keyhani
Shabaana A. Khader
Arifa S. Khan
M. Nadeem Khan
Niharika Khanna
Krystyn Kibler
Megan R. Kiedrowski
Nicolas Kieffer
Peter E. Kima
Samantha Jane King
David L. Kirchman
Thomas Kislinger
Kimberly A. Kline
Matthew D. Koci
Andrew Young Koh
Takahiko Koizumi
James B. Konopka
Karen A. Kormuth
Daniel Kornitzer
Anna D. Koromyslova
Nicole Marie Koropatkin
Anita A. Koshy
Nalinikanth Kotagiri
Susan F. Koval
Lukasz Kozubowski
Varvara Kozyreva
Oliver Kramer
Jan-Ulrich Kreft
Gopinath Krishnamoorthy
Carol A. Kumamoto
Anand Kumar
Ashwani Kumar
Binod Kumar
Nirbhay Kumar
Rolf Kümmerli
Cemil Kürekci
Kyohei Kuroda
Oliver Kurzai
Hiroyuki Kusada
Marta Kuźmińska-Bajor
Monique Lafon
Gyanu Lamichhane
Erwin Lamping
Kristin Lane
Stephanie Langel
Brian D. Lanoil
Carla E. Lanze
Timothy M. Lapara
Anders Rhod Larsen
Antje Lauer
Adi Lavy
Mark L. Lawrence
Wilkinson Lázaro
Michael Richard Lebert
Yong-Hwan Lee
Jose A. Lemos
Vanessa Leone
Valerie Le Sage
Roger C. Levesque
Min Z. Levine
Kim Lewis
Victor H. Leyva-Grado
Jianrong Li
Lin-Xi Li
Lynne Li
Ruichao Li
Xian-Zhi Li
Jean Lim
Bruno P. Lima
Hanzhi Lin
Jianfeng Lin
Yongxin Lin
Nilton Lincopan
Amelia Ryan Isis Lindsey
Zhuoren Ling
Trevor Lithgow
Xingzhong Liu
Zhou Liu
Jose L. Lopez-Ribot
Thomas Louie
Nancy G. Love
Jelena Lozo
Diannan Lu
Lenette Lu
Shirley Luckhart
Brian Michael Luna
Zhen Luo
Calman A. MacLennan
Rajat Madan
Khandaker Rayhan Mahbub
Bernardo Alfredo Mainou
Geofrey Makenga
Burkhard Malorny
Michael Manhart
Jan Mares
Kevin Maringer
Claudia N. H. Marques
Linsey Marr
Sara Marti
Rebekah M. Martin
Miguel A. Martín-Acebes
Willames M. B. S. Martins
Gislaine Martins De Oliveira
Isabelle Martin-Verstraete
Julien Massoni
Sébastien Matamoros
Rosario Mato
Jyl S. Matson
Joseph J. Mattapallil
Ben Matthews
Joshua T. Mattila
Radheshyam Maurya
Meghan May
Andrew J. McBain
Anne E. McBride
Jere W. McBride
Conall McCaughey
Mark S. McClain
Bruce A. McClane
John K. McCormick
Randy James McCreery
John T. McCrone
Elizabeth A. McDaniel
Anita K. McElroy
Lesley McGee
Gerald Michael McInerny
David J. McMillan
David N. McMurray
Peter T. Mee
Maliheh Mehrshad
Aubrey F. Mendonca
D. Scott Merrell
Carl R. Merril
Dennis W. Metzger
Bjoern Meyer
Paul A. Michels
Firas S. Midani
Alita A. Miller
Todd Miller
Ewerton Garcia de Oliveira Mima
Luciene Minarini
Yusuke Minato
Carmen Mirabelli
Maria Miragaia
Dominique Missiakas
Stefania Mitola
Tanya A. Miura
Tim Miyashiro
Kazufumi Mochizuki
Moniruzzaman Mohammad
Igor Mokrousov
Christopher Montgomery
Catherine Moore
Gonzalo Moratorio
Karen Moreau
Hiroshi Mori
Koji Mori
Kouichi Morita
Minoru Moriyama
Thomas E. Morrison
John P. Morrissey
Julie Morrissey
Kathryn Morrissey
Joachim Morschhäuser
Kenichiro Motomura
W. Scott Moye-Rowley
Elke Mühlberger
Conrad W. Mullineaux
Matthew A. Mulvey
Carol A. Munro
Peter J. Myler
Anusha Naganathan
Moon H. Nahm
Ryosuke Nakai
Teruaki Nakatsuji
Sham Nambulli
Vinay Kumar Nandicoori
Cassandra E. Nelson
Ujjwal Neogi
Jeniel E. Nett
Irene L. G. Newton
Ryan J. Newton
Stuart T. Nichol
Wright W. Nichols
Tracy L. Nicholson
William Nicholson
Paula Niewold
Angela M. Nilius
Matthew L. Nilles
Yosuke Nishimura
Angela Helen Nobbs
Ana Rita Nogueira
Steven J. Norris
Joshua D. Nosanchuk
Takuro Nunoura
Klaus Nüsslein
Austin S. Nuxoll
Torsten Ochsenreiter
Nkechi Martina Odogwu
Kyle L. O'Donnell
Kazuhiro Ogai
Knut Ohlsen
Yusuke Okazaki
Faten A. Okda
Andrew J. Olive
Antonio Oliver
Martin Olivier
Michael Olson
Teresa R. O'Meara
Andres Opazo-Capurro
Robert C. Orchard
John Osei Sekyere
Andrei L. Osterman
Martin Pabst
Christopher Paddock
Malcolm G. P. Page
John V. Paietta
Glen E. Palmer
Timothy Palzkill
John C. Panepinto
Marcus Panning
Sneh Lata Panwar
Costas C. Papagiannitsis
Daniel Paredes-Sabja
Hyunsook Park
Dane Parker
Gabriel I. Parra
Colin R. Parrish
Sally R. Partridge
Samir N. Patel
Mark E. Peeples
José Penades
Xinxia Peng
Daniel Pensinger
Mari Pent
Jakob Pernthaler
Andreas Peschel
Karin E. Peterson
Michael Petridis
Melinda M. Pettigrew
Renata Cristina Picão
Andrzej Piekarowicz
Seth H. Pincus
Ameet J. Pinto
Johann Pitout
Paul J. Planet
Mateusz Plucinski
Mircea Podar
Shawn W. Polson
Stephen J. Polyak
Vineel Kumar Pothi Reddy
Thomas Pottage
Arjun Prasad
Vibhu Prasad
Gregory P. Priebe
Firdausi Qadri
Shangshang Qin
Jianming Qiu
Maxime Québatte
Cheryl L. Quinn
Robert Andrew Quinn
Brent Race
Gireesh Rajashekara
Stuart A. Ralph
Srinivasan Ramakrishnan
Kumaran S. Ramamurthi
Sasirekha Ramani
Maria Soledad Ramirez
Joshua P. Ramsay
Tara M. Randis
Leslie Anne Rank
Xiancai Rao
Chad A. Rappleye
Philip N. Rather
Maria Angelica Rea
Michael D. Reed
Jose A. Regla-Nava
Jonathan S. Reichner
Aaron Reinke
Jyothi Rengarajan
Nilton Renno
Peter Reuther
Kelly C. Rice
Dave Richard
Amariliz Rivera
Carmen Alicia Rivera Pérez
Ian S. Roberts
Marilyn C. Roberts
D. Ashley Robinson
Libia Zulema Rodriguez Anaya
Francisco Rodriguez-Valera
Adriana E. Rosato
David A. Rosen
Helene F. Rosenberg
Isabelle Rosinski-Chupin
Benjamin Ross
Raymond R. Rowland
Claudia Rückert
Thomas A. Russo
Jenna Rychert
David A. Sack
Xavier Saelens
Chayan Kumar Saha
Paula S. Salgado
Francisco Salinas
Anastasios Samaras
James E. Samuel
Orjan Samuelsen
Derrick Samuelson
John C. Samuelson
Sarah E. Sansom
Ana Carolina Carolina de Mello Santos
Manuela Santos
Christian Santos-Medellín
Sunil D. Saroj
Michael Joseph Satlin
Yuya Sato
Timothy J. Savage
Charles A. Scanga
Connie Schmaljohn
Thomas M. Schmidt
Mirco Schmolke
Raymond Schuch
Stacey Schultz-Cherry
William R. Schwan
Julia A. Schwartzman
Alison J. Scott
Ingrid L. Scully
Anna Maria Seekatz
Einat Segev
Julie A. Segre
Ryan F. Seipke
Ranjan Sen
Karol Sestak
William M. Shafer
Rebecca S. Shapiro
Nikki W. Shariat
Amit Sharma
Ashu Sharma
Vijay K. Sharma
Thomas J. Sharpton
Ross Shaw
Timothy P. Sheahan
Alaullah Sheikh
Samuel A. Shelburne
Aimee Shen
Robert C. Shields
Shin-Ru Shih
Takaaki Shimohata
Naoya Shinzato
Andrey N. Shkoporov
Nahum Y. Shpigel
Joshua D. Shrout
David Sibley
Lynn L. Silver
Marie Simonin
Anthony P. Sinai
Amit Singh
Herman Sintim
Beate M. Slaby
Leyla Slamti
Amy Smith
Wiep Klaas Smits
Evan S. Snitkin
Fernando C. Soncini
Stephanie D. Song
Harini Sooryanarain
Daniel O. Sordelli
Joseph A. Sorg
Paul Spearman
Fabio M. Squina
Justina Stanislaw
Maja Stanojevic
Jack T. Stapleton
Michael N. Starnbach
Jacob L. Steenwyk
Stefania Stefani
William J. Steinbach
Silke Stertz
David Cole Stevens
Marc J. A. Stevens
George C. Stewart
Alison Elizabeth Stout
Daniel Straume
Jorg Stulke
Derek Sullivan
Wei Sun
Yingjie Sun
Joyce Sutcliffe
Troy C. Sutton
Yasuhiro Suzuki
Mary Hannah Swaney
Toru Takeshita
Toru Takimoto
Michal Caspi Tal
Satoshi Tamazawa
Michelle D. Tate
Tallita C. L. Tavares
Veronika Tchesnokova
Robin Tecon
Sam R. Telford
Ben Temperton
Fred C. Tenover
Akihiko Terada
Aartjan J. W. te Velthuis
Rajagowthamee Thangavel
Kevin R. Theis
Justin A. Thornton
Yun Tian
Caroline T. Tiemessen
Anna D. Tischler
Kelvin K. W. To
Richard B. Todd
Masanori Tohno
Hirokazu Toju
Mark Alexander Toleman
Andrew Tomaras
Javier Torres
Dieter M. Tourlousse
Maria Carolina Touz
Julian Trachsel
Andrej Trauner
Pankaj Trivedi
Billy Tsai
Athanassios Tsakris
Boo Shan Tseng
Claire Elizabeth Turner
Jane F. Turton
Jessie Uehling
Juan Esteban Ugalde
Gottfried Unden
Syun-Ichi Urayama
Constantin F. Urban
Blake Ushijima
David W. Ussery
Peter Valentin-Weigand
Mark van der Giezen
Peter Van der Voort
Nicole N. van der Wel
Patrick Van Dijck
Giel G. van Dooren
David van Duin
Daria Van Tyne
Arvind Varsani
José Antonio Vázquez-Boland
Kasthuri Venkateswaran
Elisa M. Vesely
Jorge E. Vidal
Rafael Vignoli
Marius Vital
Jay Vornhagen
Martin I. Voskuil
Daniel E. Voth
Slavena Vylkova
David M. Wagner
Seth T. Walk
Edward Walker
Kimberly A. Walker
Mark J. Walker
Gemma Walton
Chengming Wang
Kai Wang
Shanquan Wang
Tony Wang
Xiaoxue Wang
Yang Wang
David Morgan Ward
Emma Wear
Mary Weber
Na Wei
Tiffany Weinkopff
Brian C. Weinrick
Brian Weiss
Tao Wen
Guido Werner
Lukas Weseslindtner
Dave J. Westenberg
Edze Westra
Dawn Wetzel
Nathan John Weyand
Robert T. Wheeler
Judith M. White
Shannon Whitmer
Gregory Wiedman
Krista Rule Wigginton
Michael R. Wiley
Brian J. Wilkinson
Julia Willett
Simon Williams
Mary E. Wilson
Jeffrey H. Withey
George Wittemyer
Christiane E. Wobus
Brian Allan Wolff
Adam C. N. Wong
Thomas Wood
Stephen M. Woodcock
Michael H. Woodworth
Aniela Wozniak
Rachel A. F. Wozniak
Xianfu Wu
Lihua Xiao
Hang Xie
Chenggang Xu
Jianping Xu
Jin-Rong Xu
Kageto Yamada
Kyosuke Yamamoto
Shinji Yamasaki
S. Steve Yan
Tao Yan
Hee-Jeong Yang
Zhaomin Yang
Hui-Ling Yen
Jae-Hyuk Yu
Yunsong Yu
Joseph P. Zackular
Raffaele Zarrilli
Egija Zaura
Ahmed Zayed
Guoquan Zhang
Lianhui Zhang
Rui Zhang
Wanjiang Zhang
Wen Zhang
Xilin Zhao
Yaofeng Zhao
Beiwen Zheng
Yong Zheng
Xiaohui Zhou
Duolong Zhu
Guoqiang Zhu
Joseph M. Ziegelbauer
Stefan Zimmermann

